# Molecular mechanisms of *Saccharomyces cerevisiae* stress adaptation and programmed cell death in response to acetic acid

**DOI:** 10.3389/fmicb.2013.00033

**Published:** 2013-02-20

**Authors:** Sergio Giannattasio, Nicoletta Guaragnella, Maša Ždralević, Ersilia Marra

**Affiliations:** Istituto di Biomembrane e Bioenergetica, Consiglio Nazionale delle RicercheBari, Italy

**Keywords:** yeast, acetic acid, cell adaptation, programmed cell death, mitochondrial retrograde pathway

## Abstract

Beyond its classical biotechnological applications such as food and beverage production or as a cell factory, the yeast *Saccharomyces cerevisiae* is a valuable model organism to study fundamental mechanisms of cell response to stressful environmental changes. Acetic acid is a physiological product of yeast fermentation and it is a well-known food preservative due to its antimicrobial action. Acetic acid has recently been shown to cause yeast cell death and aging. Here we shall focus on the molecular mechanisms of *S. cerevisiae* stress adaptation and programmed cell death in response to acetic acid. We shall elaborate on the intracellular signaling pathways involved in the cross-talk of pro-survival and pro-death pathways underlying the importance of understanding fundamental aspects of yeast cell homeostasis to improve the performance of a given yeast strain in biotechnological applications.

## INTRODUCTION

Acetic acid is a stress and death inducing agent produced *en route* to alcoholic fermentation carried out by *Saccharomyces cerevisiae*. Acetic acid can have negative effects in industrial fermentation processes such as wine production, negatively affecting wine quality (Garay-Arroyo et al., 2004; Vilela-Moura et al., 2010), or lignocellulosic fermentations for bioethanol production (Klinke et al., 2004; Liu and Blaschek, 2010; Mira et al., 2010b) underpinning its biotechnological relevance. For example, acetic acid concentration in grape must and wine may vary from 4 to even 80 mM, depending on its microbial origins (Antonelli et al., 1999; Vilela-Moura et al., 2010). Acetic acid is also a food preservative and the resistance of some yeast species to this compound can be associated with food spoilage causing major economic losses in the food industries (Stratford, 2006; Fleet, 2007). Thus the elucidation of the stress resistance and cell death mechanisms induced by acetic acid in yeast can impact the design of strategies for improving fermentations or decrease the food spoilage by acetic acid resistant-yeast species.

On the other hand, a fundamental aspect of acetic acid stress response is related to the capacity of the model organism *S. cerevisiae* to cope with newly encountered environmental conditions. Yeast may adapt and survive with alternatives in their genome expression and metabolism and is one of the most thoroughly studied unicellular eukaryotes at the cellular, molecular, and genetic level due to its well-known experimental tractability (Gasch and Werner-Washburne, 2002). Evidence has also been gathered showing that *S. cerevisiae* is able to undergo a programmed cell death (PCD) process triggered by different internal and external stimuli including acetic acid (Madeo et al., 1997, 1999; Ludovico et al., 2001, 2003). Such findings provide new tools and a model for cell death research at the molecular level (Carmona-Gutierrez et al., 2010). It is of note that mortality induced by acetic acid which accumulates in the culture medium has been proposed to participate in the mechanism of chronological aging in yeast; accordingly, buffering the medium to pH 6–7 significantly extends chronological life span (Burtner et al., 2009; Weinberger et al., 2010).

In this review we elaborate on current knowledge on the mechanisms of toxicity and tolerance to acetic acid stress obtained in the model eukaryote *S. cerevisiae.*

## ACETIC ACID STRESS AND YEAST ADAPTATION

**FIGURE 1:**
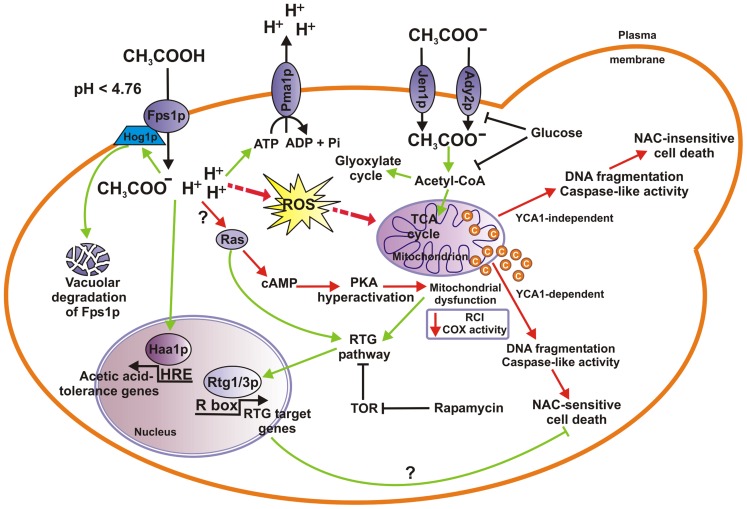
** Mechanisms of acetic acid stress response in *S. cerevisiae* cells.** When yeast cells utilize acetic acid as the sole carbon source acetate anion enters cells through either Jen1p or Ady2p monocarboxylate transporter where it is converted into acetyl-CoA which enters the TCA or the glyoxylate cycle. Both acetate transport and metabolism are inhibited by glucose. At low pH (p*K*_a_ = 4.76), in the presence of glucose, acetic acid enters cells in its undissociated form by facilitated diffusion through Fps1p aquaglyceroporin channel, where more neutral cytosolic pH causes its dissociation into acid anions and protons. Concomitant cytoplasmic acidification by protons induces the activation of the Pma1p, a plasma membrane ATPase that pumps protons out of the cell. Acetic acid challenge may activate Hog1p, a MAP-kinase involved in phosphorylation and subsequent ubiquitination, endocytosis, and final vacuolar degradation of Fps1p, and transcription factor Haa1p enabling cells to adapt to varied levels of acetic acid. On the other hand, lethal concentrations of acetic acid induce ROS accumulation, cyt *c* release and mitochondrial dysfunction, caspase-like activity increase leading eventually to cell death (AA-PCD), with chromatin condensation and nuclear DNA fragmentation occurring as PCD hallmarks. AA-PCD can occur in *YCA1*-dependent or *YCA1*-independent manner. RTG signaling pathway is proposed to be activated in certain growth conditions causing AA-PCD resistance and cell adaptation to acetic acid stress (see text for details). RTG pathway is linked to TOR and Ras signaling pathways, where the former has an inhibitory effect on Rtg1/3-dependent gene expression, and the latter enhances retrograde response. Hyperactivation of Ras–cAMP–PKA pathway can lead to mitochondrial dysfunction, ROS production and apoptosis. Cell adaptation and acetate metabolic pathways (green arrows) and AA-PCD pathways (red arrows) are shown. COX, cyt *c* oxidase; RCI, respiratory control index; ROS, reactive oxygen species; TCA, tricarboxylic acid.

Like other weak acids, acetic acid displays increased antimicrobial action at low pH (<p*K*_a_ = 4.76) in the undissociated state (Lambert and Stratford, 1999). At pH 4.5 the uncharged molecules enter cells primarily by facilitated diffusion through the Fps1p aquaglyceroporin channel (Mollapour and Piper, 2007), encounter a more neutral pH in the cytoplasm and dissociate into acetate and protons (**Figure 1**). The protons lead to cytoplasmic acidification thereby inhibiting important metabolic processes (Arneborg et al., 2000). Weak acids induce activation of the proton-translocating ATPase Pma1p in yeast plasma membrane, which pumps out the protons generated by weak acid dissociation in the cytosol in an ATP-dependent manner. This ensures maintenance of the electrochemical potential across plasma membrane regulating ion and pH balance and providing energy for nutrient uptake (Carmelo et al., 1997; Martinez-Munoz and Kane, 2008; Ullah et al., 2012).

However, the differences in weak acid toxicity appear to mirror major differences existing in the transport and metabolism of the weak acid in yeast cells. Differently from sorbic and benzoic acid, which cannot be metabolized by *S. cerevisiae* and act as membrane-damaging substances causing severe oxidative stress under aerobic conditions (Stratford and Anslow, 1998; Piper, 1999; Piper et al., 2001), acetic acid can be used as the sole carbon and energy source by *S. cerevisiae* and is not toxic under such conditions. Thus, *S. cerevisiae *cells are normally able to grow on acetic acid medium. Under this condition the weak acid is found in a dissociated form and acetate is transported across the plasma membrane either through an electroneutral proton symport transporter, encoded by *ADY2* (Casal et al., 1996; Paiva et al., 2004) or the monocarboxylate transporter encoded by *JEN1* (Casal et al., 1999). Acetate taken up by cells is converted to acetyl-CoA by one of either peroxisomal or cytosolic acetyl-CoA synthetases. Acetyl-CoA is then consumed in the glyoxylate shunt or oxidized in mitochondria through the tricarboxylic acid cycle (Vilela-Moura et al., 2008; Lee et al., 2011, and references Therein). However, typical *S. cerevisiae* cells grown on glucose cannot metabolize acetic acid due to the activation of glucose repression pathways (Rolland et al., 2002). Thus, yeast is sensitive to acetic acid stress in the presence of glucose. Acetate transport, as its metabolism, is also under glucose repression in *S. cerevisiae *but not in *Zygosaccharomyces bailii* that can metabolize acetic acid in the presence of glucose and is known for its high resistance to weak acids in glucose-containing media (Sousa et al., 1998; Rodrigues et al., 2012).

In glucose-containing media at pH 4.5 yeast cells can activate an adaptive response to weak acids, and resume to grow after a lag phase. Mechanisms of yeast adaptation to most common monocarboxylate preservatives mainly involve plasma membrane transporters and proton-translocating ATPase. Plasma membrane transporter Pdr12p, a member of ATP-binding cassette (ABC)-transporter family was strongly induced by sorbic, benzoic acid, and certain other moderately lipophilic carboxylate compounds, and to a lesser extent by acetic acid. The accumulation of Pdr12p in the plasma membrane, dependent on War1p transcription factor (see below), increases weak acid resistance mediating cellular extrusion of weak acid anion (Hatzixanthis et al., 2003; Piper, 2011).

Transcription factor Haa1p is required for a rapid yeast adaptation to acetic and propionic acids (Fernandes et al., 2005; **Figure 1**). In particular, Haa1p, directly or indirectly, specifically regulates approximately 80% of the acetic acid-induced gene expression (Mira et al., 2010a,b,c, 2011). Among the Haa1p regulon, elimination of *HRK1* and, to a lower extent, of *SAP30* gene, led to the strongest susceptibility phenotypes to acetic acid, the first gene encoding a protein kinase possibly involved in the reduction of intracellular acetate concentration and the latter encoding a component of the Rpdl3L histone deacetylase complex involved in the epigenetic regulation of yeast transcriptional response to acetic acid stress (Mira et al., 2010a). Other transcription factors which are known to orchestrate weak acid stress response in yeast including Msn2p/Msn4p and Rim101p, regulate only a few number of acetic acid-tolerance gene transcription (Schuller et al., 2004; Mira et al., 2010c; Piper, 2011).

Unlike the sorbic acid stress, in which a gain of function of Pdr12p transporter is involved in the acid resistance through *PDR12 *up-regulation, adaptation to acetic acid involves a loss of function (Mollapour et al., 2008 and references therein) of Fps1p aquaglyceroporin (**Figure 1**). Acetic acid challenge at low pH causes activation of two mitogen-activated protein (MAP) kinases, Hog1p, involved in the high-osmolarity glycerol (HOG) signaling pathway (Hohmann, 2009) and Slt2p involved in cell wall integrity pathway (Fuchs and Mylonakis, 2009). Hog1p-dependent phosphorylation of Fps1p results in its ubiquitination, endocytosis, and final degradation in the vacuole (Mollapour and Piper, 2007; Mollapour et al., 2009). Therefore, in a weak-acid specific manner, the Hog1p-directed destabilization of Fps1p eliminates the *route* for acetic acid entry into the cell, generating a resistance to varied levels of acetic acid (Piper, 2011; Zhang et al., 2011).

Such acetic acid stress response is different from hyperosmotic stress adaptation. At pH 6.8 on glucose medium cultures, very high concentrations of acetate anion (500 mM) inhibit yeast cell growth inducing a typical HOG response to sodium acetate salt stress with up-regulation of the expression of *GPD1*, encoding glycerol-3-phosphate dehydrogenase, and increased intracellular glycerol level to counteract hyperosmotic stress (Mollapour and Piper, 2006; Hohmann, 2009). At pH 4.5 a much lower acetic acid level (100 mM) is needed to cause comparable growth inhibition, with *GPD1* transcript displaying only a slight, transient induction and declining of intracellular glycerol (Mollapour and Piper, 2006). Yet, the transcription factors Gis1p and Rph1p, regulating glycerol and acetate metabolism, have been shown to function downstream of *TOR*, *RAS*/cAMP, and *AKT*/*SCH9* pathways in extending the lifespan of nutrient restricted yeast cells (Orzechowski Westholm et al., 2012).

## ACETIC ACID-INDUCED PROGRAMMED CELL DEATH

Depending on their concentrations as well as on their lipophilic moiety, weak acids may cause delay of microbial cell growth, cytostasis, or cell death (Stratford and Anslow, 1996, 1998; Piper et al., 2001). Less lipophilic acetic acid under certain conditions, compromises cell viability leading cells to death (Pinto et al., 1989; Ludovico et al., 2001).

The yeast *S. cerevisiae* undergoes a PCD process in response to lethal concentrations of acetic acid. Recent achievements in the characterization of cell components and mechanisms involved in yeast acetic acid-induced PCD (AA-PCD) are reported below (**Figure 1**).

Since the discovery of a yeast mutant exhibiting apoptosis hallmarks (Madeo et al., 1997), *S. cerevisiae* has been established as an ideal model system to study PCD pathways due to the high level of phylogenetic conservation of biochemical pathways and regulators between yeast and mammals (Carmona-Gutierrez et al., 2010). Yeast PCD shares most of the morphological and biochemical hallmarks of mammalian apoptosis, such as phosphatidylserine externalization to the outer layer of the cytoplasmic membrane, DNA fragmentation, chromatin condensation, reactive oxygen species (ROS) production as well as a pivotal role of mitochondria (Eisenberg et al., 2007; Pereira et al., 2008; Guaragnella et al., 2012).

Exponentially growing *S. cerevisiae* cells undergo PCD when exposed to 80 mM acetic acid (Ludovico et al., 2001; Giannattasio et al., 2005a). Progressive loss of viable cells is complete after 200 min from AA-PCD induction. Consistently, AA-PCD cells showed early chromatin condensation with intact plasma membrane together with ribosomal RNA degradation; nuclear DNA fragmentation ensues, with the maximum percentage at 150 min (Guaragnella et al., 2006; Ribeiro et al., 2006; Giannattasio et al., 2008; Mroczek and Kufel, 2008). The earliest event (15 min) following acetic acid challenge is ROS production, with a different role for hydrogen eroxide and superoxide anion (Guaragnella et al., 2007). Hydrogen peroxide appears to be a second messenger in AA-PCD cascade of events, as also shown by AA-PCD inhibition by ROS scavenger N-acetyl cysteine (NAC; Guaragnella et al., 2010b). ROS level *en route* to AA-PCD is modulated by the antioxidant enzymes catalase and superoxide dismutase (SOD), whose over-expression prevents and exacerbates AA-PCD, respectively (Guaragnella et al., 2008).

Mitochondria are strongly implicated in AA-PCD. Following AA-PCD induction the release of cytochrome *c* (cyt *c*) starts at 60 min and reaches a maximum at 150 min. Cyt *c* is released from intact coupled mitochondria and once in the cytosol can function both as an electron donor and a ROS scavenger. Later in AA-PCD released cyt *c* is degraded, possibly by yet unidentified proteases and mitochondria become gradually uncoupled as judged by a decrease of the respiratory control index (RCI), a collapse of the mitochondrial membrane potential, a reduction in cyt c oxidase (COX) activity and in cytochromes a–a_3_ levels (Ludovico et al., 2002; Giannattasio et al., 2008). Studies on ADP/ATP carrier, *YCA1* and cyt *c* knock-out cells have revealed that AA-PCD can also occur without cyt *c *release, but with a lower death rate compared to wild type cells (Pereira et al., 2007; Guaragnella et al., 2010a). Studies on mutant cells expressing a stable but catalytically inactive form of the protein suggested that mitochondrial cyt *c* in its reduced state modulates AA-PCD and this occurs independently on its function as an electron carrier (Guaragnella et al., 2011b).

Yeast cells have a single gene, *YCA1*, encoding a type I metacaspase that was first implicated in the execution of oxidative stress-induced PCD (Madeo et al., 2002; Wilkinson and Ramsdale, 2011). AA-PCD can occur via two alternative pathways, one dependent and the other independent of *YCA1. *The two pathways differ one from another since the latter occurs without cyt *c *release, which requires *YCA1*, and is not sensitive to the antioxidant NAC (**Figure 1**). *YCA1* participates in the AA-PCD in a manner unrelated to caspase-like activity increase which is the latest event of AA-PCD occurring at 200 min from death induction (Guaragnella et al., 2006, 2010a,b, 2011a). *YCA1* also exerts a non-death role contributing to clearance of insoluble protein aggregates over the natural yeast lifespan promoting its longevity and fitness (Lee et al., 2008, 2010).

Interestingly enough, Gup1p, an O-acyltransferase required for several cellular processes including lipid metabolism and membrane remodeling, is required for AA-PCD to occur with δ*gup1* cells dying by necrosis in response to acetic acid or in chronological aging (Tulha et al., 2012).

## THE MITOCHONDRIAL RETROGRADE PATHWAY IN YEAST CYTOPROTECTION

Acetic acid stress sensitivity of yeast cells strongly depends on the extracellular environment. Indeed, when AA-PCD is induced in yeast cells growing on glucose as carbon source at pH 3.0, it has been shown that 30 min pre-conditioning in pH 3.0 medium set by HCl prior to acetic acid administration protects *S. cerevisiae *cells from AA-PCD (Giannattasio et al., 2005a). Since acetic acid is absent in the pre-conditioning medium, the hypothesis that the Hog1p-dependent degradation of Fps1p, described in Section “Acetic Acid Stress and Yeast Adaptation,” could be involved in acid pre-conditioning (Mollapour and Piper, 2007; Mollapour et al., 2008) should be ruled out.

Instead, differently from AA-PCD cells, in acid stress-adapted cells acetic acid treatment does not cause any increase in intracellular ROS production (Giannattasio et al., 2005a; Guaragnella et al., 2007). Since mitochondria are the main source of ROS and a decline of mitochondrial function is observed *en route* to AA-PCD (Giannattasio et al., 2008), activation of mitochondrial stress response might be hypothesized under acid stress adaptation. **Figure 1** shows certain signaling pathways involved in cell response to mitochondrial dysfunction that may have a role in the cross-talk between cell death and adaptation mechanisms activated by acetic acid stress in yeast. The best characterized mechanism of cell response to mitochondrial dysfunction is the retrograde (RTG) pathway. Components and molecular details of RTG pathway have been better characterized in yeast (Butow and Avadhani, 2004; Liu and Butow, 2006). RTG-target gene expression is largely increased in cells with compromised mitochondrial function, such as cells lacking mitochondrial DNA (ρ^0^; Liao et al., 1991). Rtg1p and Rtg3p are transcription factors that interact as a heterodimer to bind target sites called R boxes (GTCAC) located in the promoter region of the RTG target genes (Jia et al., 1997). Activation of Rtg3p correlates with its partial de-phosphorylation and its translocation with Rtg1p from the cytoplasm to the nucleus (Sekito et al., 2000). Rtg2p acts upstream of the Rtg1/Rtg3p complex, being both a proximal sensor of the mitochondrial dysfunction and a transducer of mitochondrial signals controlling Rtg1/3p nuclear localization through the reversible binding with Mks1p, a negative regulator of the RTG pathway (Uren et al., 2000; Liu et al., 2003, 2005). Other positive and negative regulators of the RTG pathway include Bmh1p, Bmh2p, Grr1p, and Lst8p (Liu et al., 2001, 2003, 2005; Giannattasio et al., 2005b). Hog1p has been shown to control Rtg1/3p nuclear localization and to phosphorylate Rtg3p upon osmostress (Ruiz-Roig et al., 2012). Activation of the RTG pathway leads to up-regulation of a subset of nuclear genes whose products function in anaplerotic pathways, fatty acid oxidation, and glyoxylate cycle (Butow and Avadhani, 2004; Liu and Butow, 2006).

It is of note that the RTG pathway is linked to other signaling pathways, such as target of rapamycin (TOR) pathway, which regulates cell growth in response to nutrient availability, and it has been reported to inhibit Rtg1/3-dependent gene expression (Komeili et al., 2000). However, it is clear that these two pathways do not overlap but act in parallel to converge on Rtg1/3p (Giannattasio et al., 2005b). The RTG response is also related to the Ras–cAMP signaling pathway (Jazwinsky, 2003). The inappropriate activation of PKA can lead to the production of dysfunctional, ROS generating mitochondria, and apoptosis (Colombo et al., 1998; Lastauskiene and Citavicius, 2008; Leadsham and Gourlay, 2010; **Figure 1**). In this context, it is of note that both TOR and Ras–cAMP–PKA signaling pathways are causally involved in yeast AA-PCD (Phillips et al., 2006; Almeida et al., 2009).

Our initial results suggest that RTG-dependent signaling may be activated in response to mitochondrial dysfunction in acid-stressed *S. cerevisiae *cells grown in the low pH medium used for cell pre-conditioning. In this conditions, the gene encoding peroxisomal citrate synthase *(CIT2)*, is up-regulated in ρ^0^ cells compared to respiratory competent *ρ*^+^ cells, a hallmark of RTG-dependent transcription activation. On the contrary, RTG pathway remains inactive in response to mitochondrial dysfunction in cells grown in neutral pH medium, which are sensitive to AA-PCD induction (unpublished results). This points to a possible role of RTG pathway in AA-PCD signaling (Ždralević et al., 2012).

Mitochondrial RTG signaling occurs also in mammalian cells as a result of mtDNA mutation/deletion, oxidative stress, hypoxia, treatments with specific inhibitors of the respiratory chain or drugs (Butow and Avadhani, 2004). The signaling cascade is characterized by the activation of different nuclear transcription factors, including NF-κB which controls the transcription of a variety of target genes involved in the general stress response. In terms of pro-survival and adaptive response, the RTG-dependent signaling pathway in yeast and the NF-κB pathway active in mammalian cells appear to be involved in a conserved mechanism of cell stress response (Srinivasan et al., 2010), validating yeast as a model to study mitochondrial stress response pathways (Jazwinski and Kriete, 2012; Ždralević et al., 2012).

Even a traditional industry such as wine production is taking over the challenge of tailoring genetically customized wine-yeast strains. Market-oriented wine-yeast strains are currently being developed for the cost-competitive production of wine with minimized resource inputs, improved quality, and low environmental impact (Pretorius and Bauer, 2002). The comprehension of the complex mechanism integrating the signaling network activated by acetic acid *per se*, nutrient availability and metabolic conditions will greatly impact the improvement of both biological control of wine-spoilage microorganisms and, on the other hand, wine-yeast fermentation performances (Pretorius, 2000). With this respect, it is of note that laboratory yeast strains are unable to completely transform all the sugar in the grape must into ethanol under winemaking conditions, where multiple stresses occur simultaneously and sequentially throughout the fermentation (Mitchell et al., 2009). Post-genomic techniques and a systems biology approach will help to elucidate how the responses of wine yeasts to these stimuli differs from laboratory strains (Pizarro et al., 2007).

## Conflict of Interest Statement

The authors declare that the research was conducted in the absence of any commercial or financial relationships that could be construed as a potential conflict of interest.
